# Predicting Pulmonary Function Testing from Quantified Computed Tomography Using Machine Learning Algorithms in Patients with COPD

**DOI:** 10.3390/diagnostics9010033

**Published:** 2019-03-21

**Authors:** Joshua Gawlitza, Timo Sturm, Kai Spohrer, Thomas Henzler, Ibrahim Akin, Stefan Schönberg, Martin Borggrefe, Holger Haubenreisser, Frederik Trinkmann

**Affiliations:** 1 Institute of Clinical Radiology and Nuclear Medicine, University Medical Center Mannheim, Medical Faculty Mannheim, Heidelberg University, Theodor-Kutzer-Ufer 1-3, 68167 Mannheim, Germany; t.henzler@diagnostik-muenchen.de (T.H.); stefan.schoenberg@umm.de (S.S.); holger.haubenreisser@umm.de (H.H.); 2 Department of General Management and Information Systems, University of Mannheim, 68131 Mannheim, Germany; timo.sturm@googlemail.com (T.S.); spohrer@uni-mannheim.de (K.S.); 3 1st Department of Medicine (Cardiology, Angiology, Pulmonary and Intensive Care), University Medical Center Mannheim, Medical Faculty Mannheim, Heidelberg University, Theodor-Kutzer-Ufer 1-3, 68167 Mannheim, Germany; ibrahim.akin@umm.de (I.A.); martin.borggrefe@umm.de (M.B.); frederik.trinkmann@umm.de (F.T.); 4 DZHK (German Center for Cardiovascular Research), partner site, 68167 Mannheim, Germany; 5 Department of Biomedical Informatics of the Heinrich-Lanz-Center, University Medical Center Mannheim, Medical Faculty Mannheim, Heidelberg University, Theodor-Kutzer-Ufer 1-3, 68167 Mannheim, Germany

**Keywords:** chronic obstructive pulmonary disease, machine learning, thorax

## Abstract

Introduction: Quantitative computed tomography (qCT) is an emergent technique for diagnostics and research in patients with chronic obstructive pulmonary disease (COPD). qCT parameters demonstrate a correlation with pulmonary function tests and symptoms. However, qCT only provides anatomical, not functional, information. We evaluated five distinct, partial-machine learning-based mathematical models to predict lung function parameters from qCT values in comparison with pulmonary function tests. Methods: 75 patients with diagnosed COPD underwent body plethysmography and a dose-optimized qCT examination on a third-generation, dual-source CT with inspiration and expiration. Delta values (inspiration—expiration) were calculated afterwards. Four parameters were quantified: mean lung density, lung volume low-attenuated volume, and full width at half maximum. Five models were evaluated for best prediction: average prediction, median prediction, *k*-nearest neighbours (kNN), gradient boosting, and multilayer perceptron. Results: The lowest mean relative error (MRE) was calculated for the kNN model with 16%. Similar low MREs were found for polynomial regression as well as gradient boosting-based prediction. Other models led to higher MREs and thereby worse predictive performance. Beyond the sole MRE, distinct differences in prediction performance, dependent on the initial dataset (expiration, inspiration, delta), were found. Conclusion: Different, partially machine learning-based models allow the prediction of lung function values from static qCT parameters within a reasonable margin of error. Therefore, qCT parameters may contain more information than we currently utilize and can potentially augment standard functional lung testing.

## 1. Introduction

Chronic obstructive pulmonary disease (COPD) is a common and largely avoidable disease that is characterized by irreversible airway obstruction, attributable to inhaled noxae and particles. Therapeutic decisions in patients with COPD have largely relied on spirometry. However, given the 2017 GOLD report, spirometry is no longer valid for therapeutic decision-making. Currently, medication is chosen by clinical criteria without widespread availability of objective, quantifiable diagnostic tools [1].

Quantified computed tomography (qCT) is an emerging technique in the complex field of COPD diagnostics. First described in 1988 by Muller et al., they used standardized, visual “density masks” to quantify emphysema in patients with COPD [2]. With advances in scanner technologies and evaluation algorithms, increasing amounts of information can be gathered from non-contrast-enhanced chest CTs in COPD. For example, bronchial airway parameters were shown to correlate not only with lung function parameters but also with exacerbation rates and clinical symptoms [3,4,5]. Additionally, significant pathological findings in non-contrast-enhanced chest CT in patients with COPD, such as emphysema and air trapping, are associated with an increased mortality [6,7,8]. On the basis of this evidence, the American Thoracic Society and the European Respiratory Society proposed to evaluate routine chest CT in patients with newly diagnosed COPD [9].

In light of these findings, as well as emergent research in this field, qCT is more likely to contain additionaly, comprehensive COPD-related information than is currently understood and utilized.

Machine-based learning algorithms comprise emergent techniques to evaluate potential connections between imaging data and clinical parameters or outcomes [10,11]. Recently, several commercially available software solutions, using algorithms similar to those in stroke imaging and diagnosis, have been successfully evaluated [12]. However, the potential benefits of these methods have currently not been applied to the field of qCT in patients with COPD.

Therefore, the aim of this study was to evaluate the feasibility of predicting lung function parameters with radiomics features, acquired by non-contrast-enhanced chest qCT, in patients with COPD. Various mathematical models as well as machine learning-based algorithms were evaluated.

## 2. Methods

### 2.1. Subjects

The HIPAA-compliant study protocol, which is in accordance to the Declaration of Helsinki, was approved by our local ethics committee (2015-415M-MA).

We prospectively enrolled 75 consecutive patients with previously diagnosed COPD and a clinical indication for non-contrast-enhanced chest CT in a single center approach. Written informed consent was obtained from all patients following a full explanation of the purpose of the study and of potential risks and discomfort associated with their participation.

### 2.2. Lung Function Testing

All patients underwent whole-body plethysmography (MasterScreen® Body, CareFusion, Höchberg, Germany) which yielded the following parameters: forced expiratory volume in one second (FEV_1_), ratio of residual volume to total lung capacity (RV%TLC), and Tiffeneau index (FEV_1_/VC). Apart from RV%TLC, all plethysmographic values are represented as the percentage of the expected value, as calculated according to current ATS/ERS recommendations [13,14].

### 2.3. Computerized Tomography Examinations

A non-contrast chest scan was performed in maximum inspiration and maximum end-expiration using a third-generation dual-source CT system (Somatom FORCE, Siemens Healthineers, Forchheim, Germany) at 100 kVp with an additional tin filter behind the source for dose reduction [15,16]. In addition to a pre-imaging briefing, the patients received voice commands for optimal inspiration and expiration results. Existing medication was not withheld prior to imaging. The scan parameters were as follows: 100 kVp tube voltage, automated tube current modulation using 96 mAs at 120 kVp as reference (effective mAs = 166.5 ± 105), 0.25 s rotation time, pitch 1.2, 192 mm × 0.6 mm detector collimation. All images were reconstructed with a slice thickness of 1.5 mm, using a suitable reconstruction kernel for quantitative lung analysis (Br32) and a third-generation, iterative reconstruction technique (Adaptive Model-based Iterative reconstruction [ADMIRE], Siemens Healthineers, Germany). The reconstruction algorithm was explained in a recent publication by Gordic et al. [17]. An iterative strength level of four (out of a maximum of five) was chosen for the present study for optimum image noise, as recommended by the CT vendor for quantitative lung analysis. The average CTDI was 0.48 ± 0.19 mGy and the mean DLP 17.2 ± 6.5 mGy·cm.

### 2.4. Image Analysis

Inspiratory and expiratory datasets were analyzed using dedicated semi-automatic software (SyngoViaVB10, Pulmo3D, Siemens Healthineers, Forchheim, Germany). Lung segmentation was automated and manually revised only if necessary. Four quantitative parameters were acquired: total lung volume (volume), mean lung density (MLD), full-width-half-max (FWHM), and low attenuation volume (LAV). The LAV threshold for emphysema was set to −950 HU. This cut-off has been extensively evaluated in previous studies and strongly correlates with microscopic and gross emphysema [18,19,20]. FWHM marks the width at the half maximum of the voxel count to a specific HU value curve, representing the density distribution of the lung parenchyma. The difference of the values between inspiratory and expiratory scans were defined as delta values and were used as a distinct dataset in this evaluation. The use of this additional delta data has shown to be beneficial in previous studies [3].

### 2.5. Model Training and Evaluation

Five models were used for the prediction of lung function values: mean prediction, median prediction, polynomial regression, *k*-nearest neighbor regression (kNN), an additive regression with regression trees in XGBoost, and an artificial neural network multilayer perceptron regression (Tensorflow, Google, Mountain View, CA, USA). All breathing states (inspiration, expiration, delta) and parameters (volume, MLD, LAV and FWHM) were used as input data for every model.

**Figure 1:**
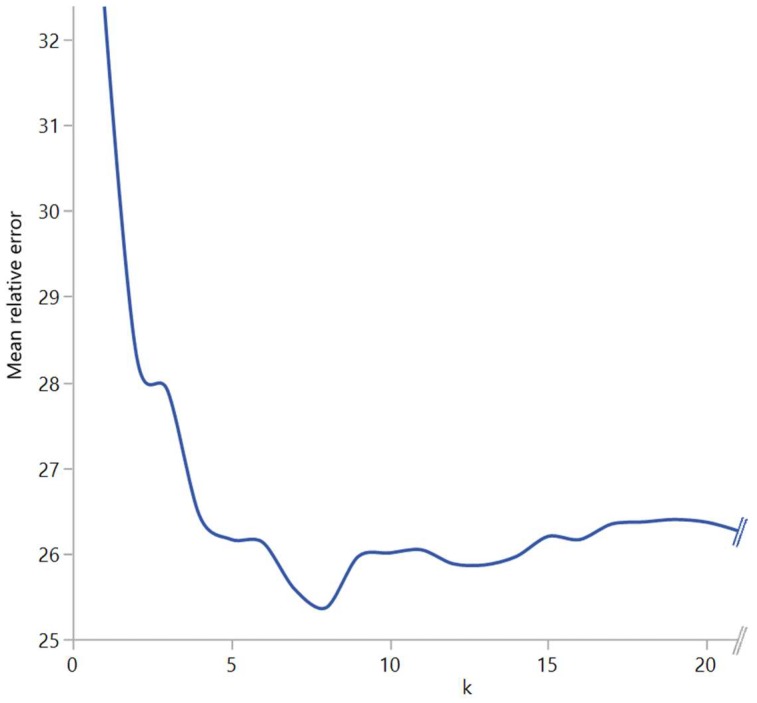
Detail of the brute force approach to find ideal *k* in *k*-nearest neighbour analysis. The mean relative error of the RV%TLC prediction is shown for various *k*s.

Mean and median prediction was performed, using target lung function values. Therefore, both methods yielded the same results for inspiration, expiration, and delta values. To find best performance, polynomial regression and kNN were performed using different degrees and k values, respectively. The polynomial regression was calculated for every degree from 1 to 10 because the performance peak had already been reached after the third degree. The kNN regression was performed for all k-values between 1 and 74 because 75 patients were included in the study. The approach of finding the ideal k and degree can be seen in Figure 1. Weighted voting based on distance was used to combine the values of the k neighbors.

XGBoost and multilayer perceptron largely remained at their default settings. For XGBoost, this meant a total number of 100 trees, with a maximal depth per tree of three and a boosting learning rate of 0.1. The multilayer perceptron had five neurons building the input layer, two hidden layers with 256 neurons each, and one output neuron.

### 2.6. Model Evaluation and Statistical Analysis

“Leave one out” cross validation was used to measure the prediction performance for all models and parameters, which has been shown to be beneficial for small cohorts [21,22]. The mean absolute and the corresponding mean relative error (MRE) were used as markers for prediction performance [23]. Both values were compared for every model, breathing state, and parameter using ANOVA and corrected by Tuckey HSD for multiple testing via dedicated software (JMP 12, SAS, Cary, CA, USA). A *p*-value of less than 0.05 was considered statistically significant.

## 3. Results

### 3.1. Data Mining

In the brute force approach to find the ideal *k* for the kNN model and the degree of the polynomial regression, respectively, different values demonstrated the best mean relative error. The degrees are shown in Table 1. These values were used in additional analysis for comparison with the performance parameters of other mathematical models.

### 3.2. Model Comparison in Prediction Performance

The actual measured values of the lung function testing, the predicted values of the individual algorithm, and the *p*-values of the ANOVA are shown in Table 2.

Significant differences in the ANOVA among both predicted and measured values were found for %FEV_1_ in all datasets (inspiration (*p* = 0.0024), expiration (*p* = 0.0078), delta (0.002)), and FEV_1_/VC in the delta dataset (*p* = 0.0005). As shown in subsequent analysis, the predicted lung function values of the median prediction model showed significant differences to all other parameters for %FEV1 in the inspiration and expiration dataset. For FEV_1_ in the delta dataset, the median prediction values only showed significant differences to the XGBoost values (difference = 8%; *p* = 0.0479). The mean difference between median prediction values and the other models for the %FEV1 was 7.4 % (mean *p* = 0.0248) in the expiration and 7.6% (mean *p* = 0.0117) in the inspiration dataset. In all three cases, the median prediction showed lower values when compared with the actual body plethysmography values as well as the other prediction models.

The ANOVA also showed a difference in variance for the FEV_1_/VC in the delta dataset. However, the neural network model-based values showed significant lower values when compared to the other models and body plethysmography results (mean difference = 7.7%; mean *p* = 0.0001). For RV/TLC, no significant differences were found in any dataset.

Overall, the predicted values from the kNN model, the polynomial regression, the mean prediction, and the XGBoost model did not show significant differences to the measured lung function values (*p*-range 0.06–1).

### 3.3. Absolute and Relative Errors of Prediction Models

With regard to the mean absolute and relative errors of the predicted lung function values, no significant difference among the used models was found. The MRE ranged between 16 and 47 percent. Overall, kNN and the polynomial regression consistently showed the lowest absolute and relative errors in relation to the measured body plethysmography values.

**Figure 2:**
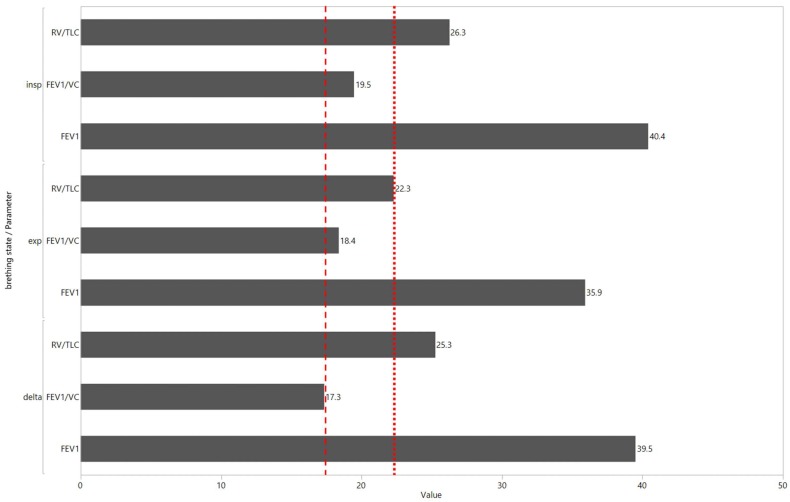
Mean relative errors of the polynomial regression for all datasets (inspiration, expiration, delta) and predicted parameters. The dotted and dashed line mark the distinct differences in prediction performance with regard to the expiration and delta scan for the FEV1/VC and RV/TLC, respectively.

Despite the lack of significant differences in the statistical analysis, there were distinct differences among the predicted lung function values of the different models. As shown in Figure 2, the MRE of the polynomial regression for RV/TLC (dotted line) was lower in the expiratory dataset (Ø 22.3) in comparison with the inspiratory (Ø 26) and delta datasets (Ø 25). This phenomenon was also noticeable for FEV_1_/VC. In this case, the lowest MRE was calculated for the delta dataset (Ø 17) in comparison with the expiratory (Ø 18) and inspiratory datasets (Ø 20), with details given in Table 3.

## 4. Discussion

This work evaluated the feasibility of predicting lung function values by parameters derived from qCT using different mathematical models. We demonstrated that this prediction can be performed within a reasonable margin of error with regard to differences in target values and prediction models. Overall, kNN and polynomial regression showed the lowest MREs in our study. As shown in previous studies, kNN and polynomial regression are suitable methods for prediction models in small sample sizes [24,25].

Our analysis found only minor significant differences in prediction performance among the evaluated models. Significant differences were shown for the median prediction in the FEV_1_. One reason for this might be that the median prediction was only based on the lung function values and was used as a baseline for the other prediction models. This explains the low standard deviation of both as well as mean and median prediction.

The XGBoost-based prediction showed the FEV_1_ in the delta values and significantly lower prediction values when compared with the other models. That only significant differences were found in the FEV_1_ seems to be consistent when looking at the global results: all prediction models performed better in the prediction of FEV_1_/VC and RV/TLC than only FEV_1_.

One explanation for this and the aforementioned significant differences might be that the FEV_1_ is an explicit dynamical parameter and the qCT, even when performed in two breathing states, contains only static information. Therefore, the TLC, VC, and the RV may be easier to predict with regard to the initial static information. This theory is supported by the different datasets (inspiration, expiration, delta), which showed different MREs with regard to the parameters. This might be attributable to the physiological meaning of the different datasets. In terms of the expiratory dataset, if the patient is in maximal expiration, the volume measured in the expiratory CT is equivalent to the RV. The is similar to the inspiratory CT, which is, at maximal inspiration, equivalent to the TLC. Similarly, if we examine the delta dataset, the subtraction of inspiratory (TLC) and expiratory (RV) data, we receive the maximal mobilizable volume, i.e., the VC. Accordingly, the various MREs seem to be consistent because the lowest MRE for the FEV_1_/VC was found in the delta dataset. This was similar to the RV/TLC, which was the lowest in the expiration dataset. Even if the differences were not statistically significant, the calculated results were in line with our physiological understanding of the different qCT datasets. Similar findings have been reported in previous studies [3,26,27]. A larger cohort might confirm our hypothesis to a significant level. In summary, this highlights the importance of an additional scan in maximal expiration, as stated by the Fleischner Society and other studies [28,29,30,31].

Most previous studies only correlated lung function values with qCT parameters but did not attempt to predict the lung function itself [3,27,29,31]. Nevertheless, Gu et al. predicted lung function values based on the emphysema acquired by qCT. They calculated MREs in the prediction of the FEV_1_% between 17% and 256% [32]. Similar to our study, the prediction of FEV_1_/VC performed better when compared with the FEV_1_% prediction. In comparison with our approach, they only used one linear prediction model and only different subdivisions of the LAV as initial prediction factors.

In our analysis, the neural-network-based prediction did not perform significantly different when compared with the other mathematical models. With regard to the rise of neural-network-based models, this seems counterintuitive [10,11]. A significant reason for this may be our sample size. As shown in previous work, the size of the initial training set is crucial for the performance of such a neural-network-based prediction model [33,34]. A larger initial dataset will likely significantly improve the performance.

Our study contained several limitations, one of which was our relatively small sample size. A larger cohort will not only improve performance in the neural-network-based model but will also improve generalizability in all models. Further, we evaluated only a limited variety of algorithms with specific settings. A more comprehensive tuning of the profound parameters in XGBoost and the neural network-based model would most likely have improved the prediction performance. Further studies should evaluate the influence of different factors, for example, tree depths, network layers, or boosting rates.

A third problem was in the initial training dataset itself. We chose a real-life dataset of patients with diagnosed COPD. With regard to the heterogeneity of COPD and its airway obstruction states, a wide standard deviation is present. Therefore, with regard to the small sample size, the models had to adapt to a large range of values. Again, especially for such a heterogenic cohort, a larger sample would be beneficial.

In summary we were able to show that different prediction models can be used to predict lung function values from qCT in patients with COPD. Therefore, the best prediction performance was most likely attributable to the sample size, which was calculated for kNN and polynomial regression. Nevertheless, the prediction performance was generally not significantly different from the other models, suggesting they can also be used for similar objectives. Therefore, computationally incomprehensive models (e.g., KNN or polynomial regression) might offer valid alternatives in lung function prediction when compared with neural networks. Further evaluations with larger sample sizes might benefit the XGBoost and neural-network-based prediction. Nevertheless, the prediction performance of the models used in this work significantly overcomes previous, one-linear regression systems using explorations [32].

Overall, the prediction of static parameters was superior when compared with the ones of dynamic parameters. Additionally, different breathing states showed different prediction performances with regard to individual parameters. In contrast to inspiration-only-based research, this underlines the need for an additional expiratory scan; further studies should take this as well as the calculated delta values into account [35].

Although traditional, inexpensive lung function testing (e.g. spirometry) is not likely to be replaced by quantified computed tomography, there might be a future application for similar prediction models. Sites without a pneumology department or additional information from a noncontrast-enhanced chest CT in outpatient care would benefit from such a prediction system because lung function changes might be recognized earlier. Further, specific pneumological testing could be carried out after these incidental findings. As seen in this proof-of-concept work, quantified computed tomography can be used to predict lung function values in COPD. The distinct differences shown between dynamic and static parameters as well as the importance of separate inspiratory, expiratory, and delta datasets should be useful for research groups for future evaluation.

## Figures and Tables

**Table 1:** *k*s and degrees with the lowest mean relative error.

*k*s and Degrees with Lowest Mean Relative Errors		
	*k* with Best MRE	Degree with Best MRE
Inspiration %FEV_1_	23	1
Inspiration %FEV_1_/VC	14	1
Inspiration RV/TLC	13	2
Expiration %FEV_1_	6	3
Expiration %FEV_1_/VC	13	2
Expiration RV/TLC	7	3
Delta %FEV_1_	7	2
Delta %FEV_1_/VC	12	1
Delta RV/TLC	8	1

FEV_1_: forced expiratory volume in one second; VC: vital capacity; RV: residual volume; TLC: total lung capacity; MRE: mean relative error.

**Table 2:** Lung function values from body plethysmography and the predicted lung function values from all algorithms used with their standard deviation.

Lung Function Values from Body Plethysmorgraphy and Predicted Lung Function Values									
		Measured Values	Mean Prediction	Median Prediction	kNN	Polynomial Regression	Neural Network	XGBoost	*p*-Value
inspiration	%FEV_1_	56 ± 6	56 ± 0.3	48 ± 0.1	56 ± 6	55 ± 13	55 ± 13	56 ± 16	0.0024
	%FEV_1_/VC	55 ± 7	56 ± 0.1	54 ± 0.1	55 ± 7	56 ± 7	56 ± 7	56 ± 10	0.732
	RV/TLC	59 ± 6	60 ± 0.2	61 ± 0.5	59 ± 6	60 ± 10	60 ± 11	59 ± 11	0.96
expiration	%FEV_1_	53 ± 12	56 ± 0.3	48 ± 0.1	53 ± 12	56 ± 14	55 ± 14	56 ± 16	0.0078
	%FEV_1_/VC	55 ± 8	56 ± 0.1	54 ± 0.1	55 ± 8	56 ± 8	56 ± 8	56 ± 11	0.6
	RV/TLC	61 ± 9	60 ± 0.2	61 ± 0.5	61 ± 9	59 ± 11	59 ± 12	59 ± 12	0.7996
delta	%FEV_1_	56 ± 13	56 ± 0.3	48 ± 0.1	56 ± 13	56 ± 14	49 ± 27	56 ± 16	0.002
	%FEV_1_/VC	55 ± 8	56 ± 0.1	54 ± 0.1	55 ± 8	56 ± 8	49 ± 25	56 ± 11	0.0005
	RV/TLC	59 ± 8	60 ± 0.2	61 ± 0.5	59 ± 8	59 ± 8	54 ± 26	58 ± 13	0.0522

Measured lung function values from the body plethysmography and predicted lung function values from the mathematical models, both with their standard deviation. For *k*-nearest neigbours and the polynomial regression, only the best performing *k*s and degrees are shown (see Table 1). The p-values of the ANOVA are shown in the right column. FEV1: forced expiratory volume in one second; VC: vital capacity; RV: residual volume; TLC: total lung capacity; kNN: *k*-nearest neighbour.

**Table 3:** Absolute and relative errors of all algorithms tested.

Absolute and Relative Errors of Predicted Lung Function Values													
		Mean Prediction		Median Prediction		kNN		Polynomial Regression		Neural Network		XGBoost	
		MAR	MRE	MAR	MRE	MAR	MRE	MAR	MRE	MAR	MRE	MAR	MRE
inspiration	%FEV_1_	21 ± 13	46 ± 43	20 ± 16	39 ± 6	19 ± 13	43 ± 39	19 ± 13	40 ± 37	20 ± 13	41 ± 34	22 ± 18	47 ± 50
	%FEV_1_/VC	12 ± 8	22 ± 17	11 ± 8	21 ± 12	9 ± 8	17 ± 15	10 ± 7	19 ± 15	12 ± 8	22 ± 17	10 ± 8	19 ± 16
	RV/TLC	14 ± 10	30 ± 40	14 ± 10	31 ± 42	13 ± 9	28 ± 38	12 ± 8	26 ± 36	13 ± 9	27 ± 39	14 ± 12	31 ± 46
expiration	%FEV_1_	21 ± 13	46 ± 43	20 ± 16	39 ± 6	18 ± 15	36 ± 37	17 ± 13	36 ± 37	19 ± 14	41 ± 36	22 ± 16	46 ± 45
	%FEV_1_/VC	12 ± 8	22 ± 17	11 ± 8	21 ± 12	9 ± 8	16 ± 14	10 ± 7	18 ± 15	12 ± 8	22 ± 17	10 ± 8	20 ± 17
	RV/TLC	14 ± 10	30 ± 40	14 ± 10	31 ± 42	11 ± 9	25 ± 31	11 ± 8	22 ± 28	11 ± 8	22 ± 25	12 ± 9	24 ± 26
delta	%FEV_1_	21 ± 13	46 ± 43	20 ± 16	39 ± 6	18 ± 15	39 ± 42	19 ± 15	40 ± 37	21 ± 19	38 ± 35	18 ± 15	40 ± 46
	%FEV_1_/VC	12 ± 8	22 ± 17	11 ± 8	21 ± 12	9 ± 8	16 ± 14	9 ± 8	17 ± 15	20 ± 16	35 ± 25	9 ± 8	17 ± 16
	RV/TLC	14 ± 10	30 ± 40	14 ± 10	31 ± 42	12 ± 9	25 ± 30	12 ± 10	25 ± 30	26 ± 22	47 ± 49	14 ± 12	29 ± 34

Mean absolute error (MAR) and the mean relative error (MRE) of the predicted lung function values. The MAR is given as the amount of the absolute error needed to compensate the negative signs. FEV1: forced expiratory volume in one second; VC: vital capacity; RV: residual volume; TLC: total lung capacity; kNN: *k-*nearest neighbour; MRE: mean relative error; MAR: mean absolute error.

## Abbreviations

COPD	chronic obstructive pulmonary disease
FEV1	forced expiratory volume at one second
kNN	*k*-nearest neighbour
MRE	mean relative error
qCT	quantified computed tomography
RV	residual volume
TLC	total lung capacity
VC	vital capacity
